# Molecular characterization and B-cell epitope analysis of the TSP11 gene in *Echinococcus* infection strains from Yunnan Province

**DOI:** 10.1017/S0031182024000726

**Published:** 2024-09

**Authors:** Qian Xu, Zhengqing Wang, Jinrong Zi, Xuan Cai, Fangwei Wu, Benfu Li, Jia Peng, Jianxiong Li, Xinliu Yan, Ying Dong, Yaming Yang

**Affiliations:** Yunnan Institute of Parasitic Diseases, Puer, 665000, China

**Keywords:** B-cell epitope, *Echinococcus*, *E*. *granulosus*, genotype, TSP11 gene, Yunnan

## Abstract

This study investigates the molecular intricacies of the transmembrane protein TSP11 gene in *Echinococcus* strains isolated from livestock and patients in Yunnan Province afflicted with *Echinococcus granulosus (E. granulosus)* between 2016 and 2020. Gene typing analysis of the ND1 gene revealed the presence of the G1 type, G5 type and untyped strains, constituting 52.4, 38.1 and 9.5%, respectively. The analysis of 42 DNA sequences has revealed 24 novel single nucleotide polymorphic sites, delineating 11 haplotypes, all of which were of the mutant type. Importantly, there were no variations observed in mutation sites or haplotypes in any of the hosts. The total length of the TSP11 gene's 4 exons is 762 bp, encoding 254 amino acids. Our analysis posits the existence of 6 potential B-cell antigenic epitopes within TSP11, specifically at positions 49-KSN-51, 139-GKRG-142, 162-DNG-164, 169-NGS-171, 185-DS-186 and 231-PPRFTN-236. Notably, these epitopes exhibit consistent presence among various intermediate hosts and haplotypes. However, further validation is imperative to ascertain their viability as diagnostic antigens for *E. granulosus* in the Yunnan Province.

## Graphical abstract

**Figure:**
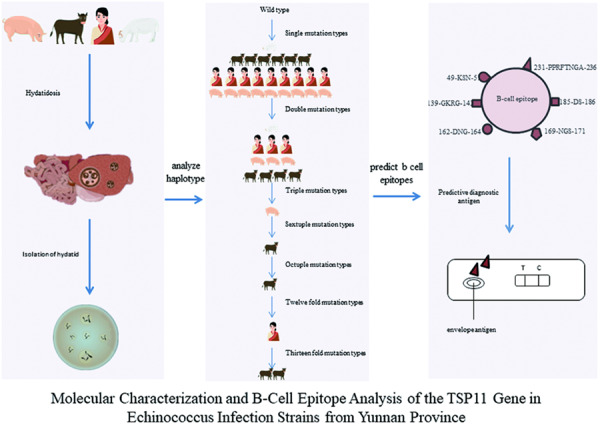

## Introduction

Echinococcosis, also known as hydatid disease, is a persistent cystic zoonotic parasitic disease that affects humans and both domestic and wild odd-toed ungulates. It is caused by the larval stage of the dog tapeworm, a member of the *Echinococcus*, family Taeniidae, specifically *E. granulosus* (Chi, 2015).

Designated by the World Health Organization as one of the 20 easily neglected tropical diseases, *E. granulosus* is regionally endemic throughout Europe, North and East Africa, Central Asia, the Middle East, Central and South America and Australia (World Health Organization, 2021; Hogea *et al*., 2024), it currently affects approximately 3 million patients with this disease, leading to an estimated annual economic loss of 760 million USD. In the livestock industry, the infection of domestic animals by the larval stage of *Echinococcus* results in economic losses exceeding 3 billion USD annually due to reduced weight and lower fertility (Budke *et al*., 2006; Otero-Abad and Torgerson, 2013; Oian et al., 2017).

China bears one of the highest incidences of hydatid disease, with primary endemic areas including Xinjiang, Sichuan, Qinghai, Tibet, Gansu, Ningxia, Inner Mongolia and semi-agricultural and semi-pastoral regions (Sivalingam and Shepherd, 2012). Incomplete statistics indicate that by the end of 2016, over 368 counties (districts) in China had been affected by hydatid disease, with an incidence rate among the population ranging from 0.6 to 4.5%, totalling approximately 170 000 patients (Kolaskar and Tongaonkar, 1990; Jiang, 2002). Hydatid disease constitutes 40% of the global burden (Wu, 2017). In Yunnan Province, *Echinococcus* endemic areas are concentrated primarily west of 25°N latitude, in regions highly affected by hydatid disease, including Ganzi in Sichuan, Changdu in Tibet and others (Qiu *et al*., 2000; Huang *et al*., 2012; Lei and Wang, 2012). Notably, counties (districts) such as Deqen and Dali exhibit an incidence rate of 0.06% among the population, with all infections identified as *E. granulosus*. However, the Diqing region also demonstrates high infection rates among animal hosts, indicating a potential risk of natural focal transmission (Li *et al*., 2019; Li *et al*., 2020).

Mitigating the disease burden of human echinococcosis involves both effective patient treatment and accurate diagnoses. Presently, the primary treatment for hydatid disease involves the surgical excision of lesion tissue and chemotherapy (Wen *et al*., 2015). While surgical intervention can promptly alleviate the harm caused by hydatid disease, it is associated with high recurrence rates and numerous postoperative complications (Guo *et al*., 2019), necessitating adjunctive drug therapy (Aghayev, 2016). Very few adverse events have been reported by treatment with albendazole, however, even a single dose treatment (for empirical or seasonal use) of albendazole (400 mg) could cause acute liver toxicity in adult patients (Chai *et al*., 2021).

Conversely, diagnostic methods reliant on imaging technology are not conducive to clear diagnoses of early-stage, non-cystic patients with echinococcosis (Craig *et al*., 2007). Consequently, immunodiagnostic tools have gained widespread use in recent years for epidemiological screening and early diagnosis of echinococcosis (Li and Gao, 2017). Nevertheless, the antigen employed for enzyme-linked immunosorbent assay (ELISA) detection plates typically comprises echinococcosis cyst fluid. The complexity of protein components in the cyst fluid compromises the accuracy of detecting *Echinococcus* due to cross-reactivity with other tapeworms of the family Diplotriaenidae. Therefore, selecting specific and highly sensitive coating antigens has become a focal point of current research (Chow *et al*., 2004; Liu *et al*., 2015; Xu *et al*., 2018). Notably, the 4-transmembrane protein (TSP11) on the surface of *Echinococcus*, identified for its pivotal role in stimulating the host's acquired immune response, serves as a specific marker for echinococcosis infection (Wang *et al*., 2017). This current study aimed to analyse the polymorphism of the TSP11 gene distributed in Yunnan and its surrounding areas and investigation of B antigenic determinant cluster of this protein for use as a diagnostic antigen.

## Materials and methods

### Study sample

Throughout the implementation of the ‘Yunnan Province Hydatid Disease Monitoring Program’ from 2016 to 2020, post-slaughter inspections and parasitic infection checks were carried out on the organs of livestock (hosts) including cattle, pigs and sheep, which displayed cysts, cystic formations or nodules. Following slaughter, we collected organ tissues with cystic masses and fluid from lesions for parasitic infection examination. The samples encompassed single-cystic, multi-vesicular and collapsed internal cyst types of hepatic hydatid disease with an average cyst diameter >5 cm. Additionally, various types of hepatic hydatid with an average cyst diameter <5 cm, situated in the first or second porta hepatis and likely to cause severe complications (such as obstructive jaundice, portal hypertension, Budd–Chiari syndrome) were included. We also considered various types of hepatic hydatid disease where drug adverse reactions were significant (hepatorenal dysfunction and other detrimental side effects), or patients struggled to adhere to medication, or the cyst continued to enlarge after more than 6 months of drug treatment. In these cases, tissues from surgically removed cystic masses and fluid from lesions were gathered for parasitic infection examination (National Health Commission of the People's Republic of China, 2017).

### Microscopic confirmation of echinococcosis infection and its genotyping

The collected tissues from affected organs and fluid from lesions underwent immediate optical microscopy (10–40×) to identify *Echinococcus* cyst walls, daughter cysts, protoscoleces or small hooks (Zhu and Su, 2019).

For samples testing positive for *E. granulosus* infection, we further sequenced the ND1 gene to identify the genotype. PCR amplification of the ND1 gene for types 1 and 5 utilized primers designed with reference sequences MN199128.1 (https://www.ncbi.nlm.nih.gov/nuccore/MN199128.1) and KY766908.1 (https://www.ncbi.nlm.nih.gov/nuccore/KY766908.1) (Table 1) (Li *et al*., 2023).

**Table 1.** Primer sequence and other information

Gene name	Primer name	Sequence (5’-3’)	Length of fragment/bp	Amplification region
TSP11	TSP11-F1	TGTTTTCTATTTTTACCCACTAGAT	1230	85 417–86 646
	TSP11-R1	TTTAAAGTTAATTAGGAGAAGGACG		
	TSP11-F2	TATTAATCCAGCTTTAAGGTCATTT	1021	85 555–86 575
	TSP11-R2	TATTTGTCCAGATAGGATTTAATGG		
NAD1	NAD1-F1	TGTTACTTGTTACTCCATTGTCT	1486	6869–8354
	NAD1-R1	TATCAAAGTAACCTGCTATGCAG		
	NAD1-F2	TTTAAGTTGGGTGGTGATTA	1232	6997–8228
	NAD1-R2	TTGAAGTTAACAGCATCACG		

### Design of primers for *Echinococcus* TSP11 gene

The primers for amplifying the *Echinococcus* TSP11 gene were designed based on the reference sequence (XP_024352489.1) ((Huang *et al*., 2017)) (Table 1).

### *Echinococcus* DNA extraction and nested PCR amplification of ND1 and TSP11 gene

Approximately 20 mg of tissue exhibiting cyst-like changes was excised from the affected organs and cut into 5–10 mm fragments. DNA extraction of *Echinococcus* was conducted following the guidelines of the DNA extraction kit, and the extracted DNA was stored at −20°C for subsequent use.

The nested PCR amplification for NAD1 and TSP11 genes involved 4 reaction systems, each comprising DNA template (2.5 μL), 2 × Taq enzyme (14.0 μL), primer forward and reverse (10 μmol /L each, 0.7 μL), and ddH_2_O (7.1 μL). The PCR reaction conditions were set at 95°C for 5 min, followed by 35 cycles of 95°C for 30 sec, 55°C for 45 sec and 72°C for 1 min 30 sec, concluding with 72°C for 10 min (Han and Gao, 2020). The second-round amplification products were visualized through 2% agarose gel electrophoresis, and the resulting products were sent to Guangzhou Tianyi Huiyuan Genetic Technology Co., Ltd., for bidirectional sequencing.

### TSP11 gene polymorphism and evolutionary relationships of sequences from different host sources

The PCR product sequencing results were compiled using DNAStar 11.0 or BioEdit 7.2.5 software to generate DNA sequences for the NAD1 and TSP11 genes. The organized sequences of 2 genes were individually analysed using BLAST, comparing them against the reference sequences MN199128.1 and XP_024352489.1. The query coverage and identity were examined, and when both query coverage and identity exceeded 98%, it was indicative that the organized sequencing sequences represented the target sequences. A match of both coverage (Query cover) and similarity (Identifies) exceeding 98% confirmed the compiled sequences as the target sequences. The coding DNA sequences (CDS) for the 4 exons encoding the TSP11 gene were concatenated from the 5′ to 3′ end (Xu *et al*., 2020). Amino acid sequences were deduced using MEGA 7.0.26 software for TSP11 and the alignment file. DnaSP 5.10 software was employed to identify haplotypes, single-nucleotide polymorphism (SNP) sites, and their mutation types (synonymous/non-synonymous) for the 4 exons of the TSP11 gene. Expected heterozygosity (He) and nucleotide diversity (π) were calculated for each haplotype (Dong *et al*., 2019 and Xu *et al*., 2020). All base substitutions were verified by examining the sequencing peak charts. Network 10.0 software was used to create intermediate network evolutionary diagrams for each haplotype.

### Prediction of B-cell antigenic determinants of different haplotypes of the TSP11 gene

B-cell epitopes of TSP11 were predicted using the IEDB online platform (https://www.iedb.org/) and the ‘Protean’ module of DNAStar 11.0. Comparative analysis of different hydrophilicity plots (Ponomarenko *et al*., 2011), accessibility (Allcorn and Martin, 2002), flexibility (Li *et al*., 2016), antigenicity (Resende *et al*., 2012) and *β*-turn regions among the amino acid chains of different TSP11 haplotypes (Hu *et al*., 2014) was conducted. Parameters such as hydrophilicity, antigenic index, flexibility and *β*-turn were assessed to identify common B-cell antigenic epitopes among different intermediate hosts and haplotypes. Points with the highest local average hydrophilicity were often situated at or adjacent to the antigenic determinant clusters (epitopes). Surface accessibility prediction considered the likelihood of amino acid residues in the antigen coming into contact with corresponding antibodies or solvent molecules. Polar amino acids, more likely to be exposed on the protein surface, were deemed probable components of antigenic epitopes. Amino acid residues with high activity represented flexible sites likely to form antigenic epitopes. The *β*-turn region and structurally loose, prominently exposed, deformable and twisted areas in irregularly coiled regions were identified as potential antigenic epitope regions exposed on the protein surface that could easily bind to antibodies.

### Statistical analysis

Establishing a counting database within Excel software, we conducted chi-square tests to analyse the distribution variances among intermediate hosts exhibiting different genotypes of *Echinococcus*, nucleotide peptides of the TSP11 gene, and disparities in haplotype detection rates across diverse intermediate hosts, maintaining a significance level of *α* = 0.05.

## Results

### Sample collection sites and *Echinococcus* infection

**Figure 1.**
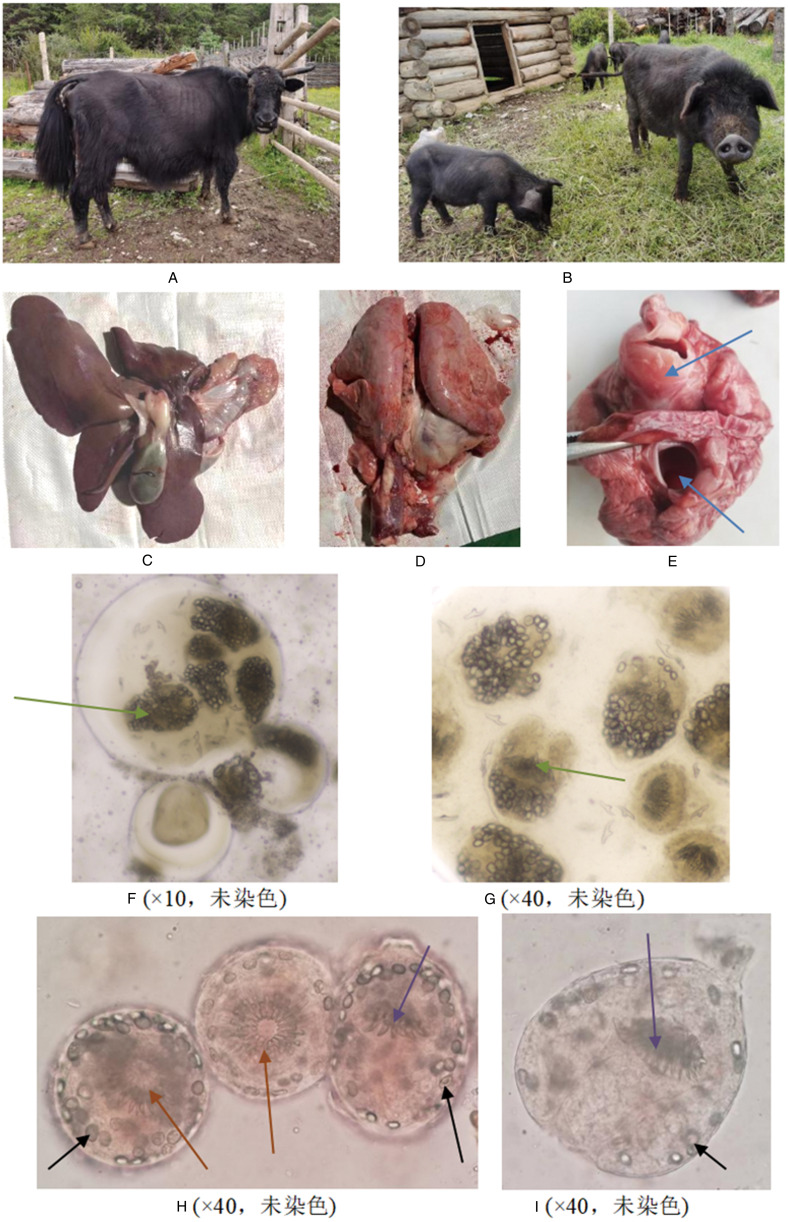
Intermediate host, diseased organ, protocephalic segment of Echinococcus granulosus Note: a: Yak from Weixi County, Diqing Prefecture; b: Diqing Tibetan pig; c: the liver; d: lungs; e: diseased part of the lung (arrow points to the diseased part); f, g: hydatid (40X); h, i: protoscolex (10X)

Between 2016 and 2020, a comprehensive total of 42 samples of visceral tissues from *Echinococcus* were meticulously collected and subjected to rigorous testing. These samples comprised 13 of human origin (4 from Jianchuan County, 4 from Ganzi Prefecture, 3 from Yulong County and 2 from Weixi County), 13 sourced from pigs (5 from Daguan County, 3 from Eryuan County, 3 from Lushui City, 1 from Shangri-La and 1 from Weixi), 14 originating from cattle (as illustrated in Fig. 1a, b) (13 from Shangri-La and 1 from Weixi) and 2 derived from sheep (1 from Weixi and 1 from Honghe). These samples encompassed 37 instances of diseased liver tissues and 6 cases of diseased lung tissues (as depicted in Figs 1c–e) (refer to Supplementary Material 1 for further details).

*Echinococcus* was detected in all 42 samples of infected tissues (Fig. 1f–i). Under microscopic examination, structures with double-layered cyst walls enclosing fluid and sand-like bodies were observed. The inner layer of the cyst wall was pink, and the cyst fluid was either transparent or slightly turbid. Small hooks floated in the fluid, and internally, elliptical or circular structures resembling ‘original heads’ were observed, which were invaginated and contracted (Fig. 1f, g, green arrows). Inside the ‘original heads,’ brown elliptical structures were identified as calcareous corpuscles (Fig. 1h, i, black arrows). The wheel-shaped structure represented a sucker (Fig. 1h, brown arrows), and the bushy structure featured small hooks (Fig. 1h, i, purple arrows).

### Sequencing of gene PCR amplification products

**Figure 2.**
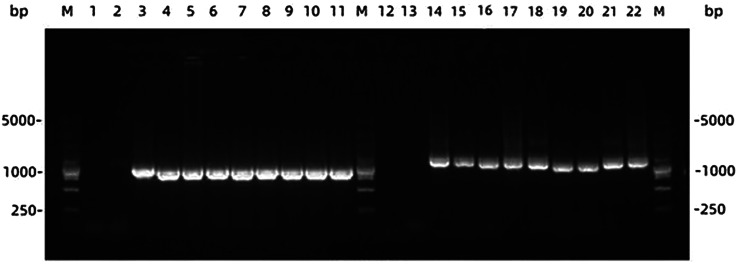
Electrophoresis Map of PCR Amplified Products of TSP11 Gene and ND1 Gene in the Pathological Organs of Echinococcus granulosus Note: M: DNA marker; 1. 2: Negative control of the first and second rounds of PCR of TSP11 gene; 3: TSP11 gene PCR positive control; 12. 13: Negative control of ND1 gene in the first and second rounds of PCR; 14: ND1 gene positive control; 4. 5, 6, 7, 8, 9, 10: amplification products of TSP11 gene; 16. 17, 18, 19, 20, 21, 22: amplification products of ND1 gene

PCR amplification of TSP11 and ND1 genes produced target bands of approximately 1021 and 1232 bp respectively (Fig. 2).

42 ND1 Gene Sequences of the 42 ND1 genes exhibited greater than 98% similarity with the reference sequences MN199128.1 and KY766908.1. Among these, the ND1 gene DNA sequences of 22 samples were identical to MN199128.1, with a length of 894 bp, classifying them as Type 1. 16 samples exhibited ND1 gene DNA sequences identical to KY766908.1, also with a length of 894 bp, categorizing them as Type 5. For the remaining 4 samples, the similarity to both MN199128.1 and KY766908.1 did not reach 100%, designating them as an undefined genotype (Table 2).

**Table 2.** Interspecific difference of ND1 gene

Order	Somatotype (According to ND1)	intermediate host				
		Person (*n* = 13)	Pig (*n* = 13)	Cow (*n* = 14)	Sheep (*n* = 2)	p
1	G1 (*n* = 22, 52.4%)	10 (45.4)	4 (18.2)	8 (36.4)	0	0.045
2	G5 (*n* = 16, 38.1%)	2 (12.5)	8 (50.0)	5 (31.3)	1 (6.2)	0.083
3	Undefined type (*n* = 4, 9.5%)	1 (25.0)	1 (25.0)	1 (25.0)	1 (25.0)	0.0428

In all, 3 distinct genotypes were identified: Type 1, Type 5 and undefined, constituting 52.4, 38.1 and 9.5% of the total, respectively. While all 3 genotypes were detectable across intermediate hosts including humans, pigs, cattle and sheep, the discrepancies in their detection rates did not reach statistical significance (*P* = 0.045, *P* = 0.083 and *P* = 0.0428) (refer to Table 2).

### Nucleotide diversity of TSP11 gene CDS sequences

Sequencing the TSP11 gene from the 42 *Echinococcus* samples resulted in complete coding DNA sequences (CDS), each comprising 4 exons with a length of 762 bp. These sequences exhibited greater than 98% similarity with the reference sequence XP_024352489.1. The nucleotide diversity index (π) was calculated to be 0.00413. There were 24 polymorphic sites, with the most frequent biallelic site being c.127 (42.9%, 18/42), and the minor allele frequency (MAF) for c.192 was 26.2% (11/42). There were 8 singleton variable sites and 16 parsimony informative sites (2 variants). Among the 24 polymorphic sites, 66.7% (16/24) were located at the third base of amino acid codons, and only 6.25% (1/16) of base substitutions resulted in amino acid variations. The proportions for the second and first base positions were 8.3% (2/24) and 25.0% (6/24), respectively (Table 3). The detection rates of these 24 polymorphic sites in sequences from different intermediate hosts such as humans, pigs, cattle and sheep showed no statistically significant differences.

**Table 3.** Single nucleotide polymorphism in the TSP11 gene of *Echinococcus granulos*

Order	Loci	Alleles	Coding	Amino acid variation	Frequency	intermediate host				P
					No. of all CDSs (*n* = 42,%)	Person (*n* = 13,%)	Pig (*n* = 13,%)	Cow (*n* = 14,%)	Sheep (*n* = 2,%)	
1	c.46	G > A	GCA/*A*CA	A16T	3 (7.1)	1 (7.7)	0	2 (14.2)	0	1.000
2	c.64	G > C	GGG/*C*GG	G22R	1 (2.4)	0	0	1 (7.1)	0	/
3	c.91	G > C	GTG/*C*TG	V31L	4 (9.5)	1 (7.7)	0	3 (21.4)	0	0.596
4	c.97	T > C	TTT/*C*TT	F33L	4 (9.5)	1 (7.7)	0	3 (21.4)	0	0.596
5	c.127	A > G	ATT/*G*TT	L43V	37 (88.1)	12	13 (100)	10 (71.4)	2 (100)	0.172
6	c.192	G > A	GGG/GG*A*	G64G	11 (26.2)	1 (7.7)	4 (30.8)	5 (11.9)	1 (50)	0.258
7	c.229	T > A	TTC/*A*TT	F77L	3 (7.1)	1 (7.7)	0	2 (14.2)	0	1.000
8	c.231	C > T	TTC/AT*T*	F77L	3 (7.1)	1 (7.7)	0	2 (14.2)	0	1.000
9	c.234	T > C	CTT/CT*C*	L78L	1 (2.4)	1 (7.7)	0	0	0	/
10	c.249	G > A	TCG/TC*A*	S83S	4 (9.5)	1 (7.7)	0	3 (21.4)	0	0.596
11	c.252	T > C	ATT/AT*C*	I84I	3 (7.1)	1 (7.7)	0	2 (14.2)	0	1.000
12	c.333	A > G	GCA/GT*G*	A111A	1 (2.4)	1 (7.7)	0	0	0	/
13	c.492	C > T	GGC/GG*T*	G164G	4 (9.5)	1 (7.7)	0	3 (21.4)	0	0.596
14	c.551	T > C	GTC/G*C*C	V184A	3 (7.1)	1 (7.7)	0	2 (14.2)	0	1.000
15	c.582	C > T	TTC/TT*T*	F194F	1 (2.4)	0	0	1 (7.1)	0	/
16	c.600	A > G	AAA/AA*G*	K200K	1 (2.4)	0	0	1 (7.1)	0	/
17	c.606	C > T	TGC/TG*T*	C202C	1 (2.4)	0	1 (7.7)	0	0	/
18	c.672	C > T	TGC/TG*T*	C224C	4 (9.5)	1 (7.7)	0	3 (21.4)	0	0.596
19	c.729	A > C	CTA/CT*C*	L243L	3 (7.1)	1 (7.7)	0	2 (14.2)	0	1.000
20	c.741	A > C	GTA/GT*C*	V247V	1 (2.4)	0	0	1 (7.1)	0	/
21	c.744	C > T	AGC/AG*T*	S248S	4 (9.5)	1 (7.7)	0	3 (21.4)	0	0.596
22	c.747	C > T	TCC/TC*T*	S249S	3 (7.1)	1 (7.7)	0	2 (14.2)	0	1.000
23	c.758	A > C	TAT/T*C*T	Y253S	1 (2.4)	0	0	1 (7.1)	0	/
24	c.759	T > C	TAT/TA*C*	Y253Y	4 (9.5)	1 (7.7)	0	3 (21.4)	0	0.596

### Multiple mutations and evolutionary analysis of TSP11 gene CDS sequences

The alignment of the 42 TSP11 gene CDS sequences with the reference sequence (XP_024352489.1) revealed the presence of 11 haplotypes. Haplotype Hap_1 perfectly matched the reference sequence, with the remaining 10 haplotypes (Hap_2 to Hap_11) representing mutated forms of the reference sequence (XP_024352489.1), resulting in expected heterozygosity (He) of 0.6622. Among them, Hap_2 had the highest frequency (57%, 24/42), followed by Hap_3 (19%, 8/42) and Hap_8 (4.8%, 2/42), with the remaining haplotypes each accounting for (2.4% 1/42) (Table 4). The mildest mutations were observed in Hap_2 (57%, 24/42) and Hap_9 (2.4%, 1/42), while the most intense mutation was observed in Hap_8 ( 4.8%, 2/42). Hap_4, Hap_5 and Hap_11 were exclusive to human samples, Hap_6 was only found in pig samples, and Hap_7, Hap_8, Hap_9, Hap_10 and Hap_12 were solely present in cattle samples. Both Hap_2 and Hap_3 were detected in samples from humans, pigs, cattle and sheep, with no statistically significant differences among the 4 groups (*P* = 0.197 and *P* = 0.387) (Table 4).

**Table 4.** Different haplotypes having mutations in *TSP11*gene

	Multiple mutants of different degrees		Frequency	intermediate host				
Haplotype	Type	Multiplicity	No. of all CDSs	person (*n* = 13, 31.0%)	pig (*n* = 13, 31.0%)	cow (*n* = 14, 33.3%)	sheep (*n* = 2, 4.8%)	p
			(*n* = 42, 100%)					
Hap_2	L43V	1	24 (57)	9 (69.2)	9 (69.2)	5 (35.7)	1 (50)	0.197
Hap_9	G64G	1	1 (2.4)	0	0	1 (7.1)	0	/
Hap_3	L43V/G64G	2	8 (19)	1 (7.7)	3 (23.1)	3 (21.4)	1 (50)	0.387
Hap_4	L43V/L78L	2	1 (2.4)	1 (7.7)	0	0	0	/
Hap_5	L43V/A111A	2	1 (2.4)	1 (7.7)	0	0	0	/
Hap_12	L43V/K200K	2	1 (2.4)	0	0	1 (7.1)	0	/
Hap_6	L43V/G64G/C202C	3	1 (2.4)	0	1 (7.7)	0	0	/
Hap_7	G22R/L43V/F194F/V247V/Y253S	6	1 (2.4)	0	0	1 (7.1)	0	/
Hap_10	V31L/F33L/S83S/G164G/C224C/S248S/S249S/Y253Y	8	1 (2.4)	0	0	1 (7.1)	0	/
Hap_11	A16T/V31L/F33L/F77L/S83S/ I84I/G164G/V184A/C224C/L243L/S248S/Y253Y	12	1 (2.4)	1 (7.7)	0	0	0	/
Hap_8	A16T/V31L/F33L/F77L/S83S/ I84I/G164G/V184A/C224C/L243L/S248S/S249S/Y254Y	13	2 (4.8)	0	0	2 (14.3)	0	/

**Figure 3.**
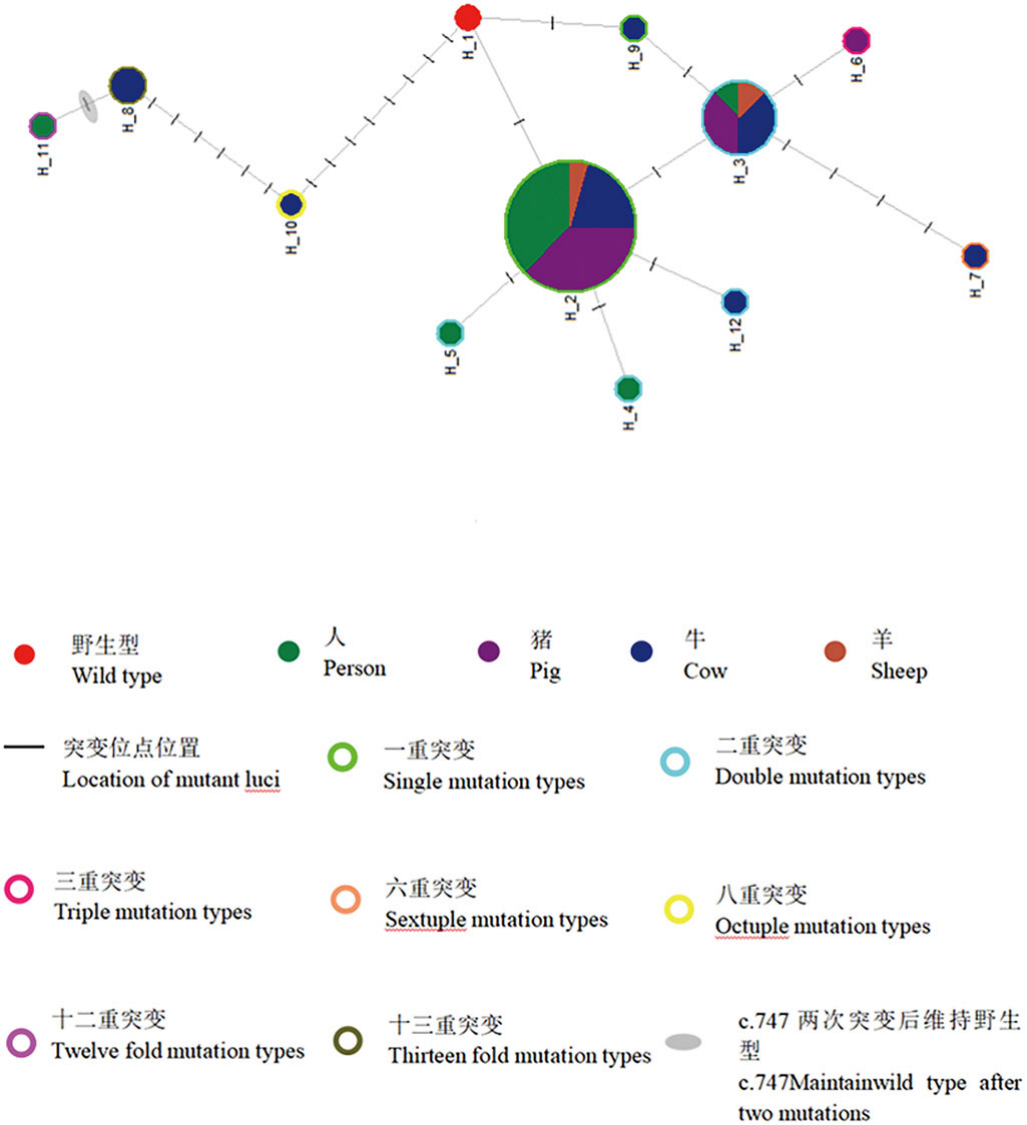
Evolutionary network of haplotype of TSP11 gene in the diseased organs of *Echinococcus granulosus*. Note: The size of the circle is proportional to the number of isolates showing a particular haplotype; lines represent evolutional steps connecting haplotypes.

The network diagram illustrated that the 11 haplotypes (Hap_2 to Hap_12) evolved from the reference sequence (XP_024352489.1) (Hap_1) through 1 mutation (Hap_2, Hap_9), progressing to 2 mutations (Hap_3, Hap_4, Hap_5, Hap_12), 3 mutations (Hap_6), 6 mutations (Hap_7), 8 mutations (Hap_10), 12 mutations (Hap_11) and 13 mutations (Hap_8). Notably, Hap_8 and Hap_11 shared the c.229 and c.231 positions, representing the same protein F77L, counted as 1 mutation in Table 4. Additionally, Hap_11 had 2 mutations at position c.747, reverting to the wild-type allele S (S249), making the joint mutation multiplicity higher in Hap_8 and Hap_11 than indicated in Table 4 (Fig. 3).

### Prediction of B-cell antigen precursors in the amino acid chain of the TSP11 gene

The translation of the 11 haplotypes (Hap_2 to Hap_12) of the TSP11 gene CDS into amino acid chains, followed by prediction using IEBD and DNA Star software, revealed 5 and 10 B-cell antigenic determinant clusters, respectively (Table 5). These clusters had lengths ranging from 3 to 40 amino acids, with an average hydrophilicity value of 0.460 (see Supplementary Material 2 for detailed results). The predicted regions, such as 49-KSN-51, 139-GKRG-142, 162-DNG-164, 169-NGS-171, 185-DS-186 and 231-PPRFTN-236, were identified as 6 conserved B-cell antigenic epitopes among the 11 amino acid chains (Table 5).

**Table 5.** Prediction of antigen determinant of TSP11 gene

Order	Predictive determinant of IEBD^a^	Predictive determinant of DNA Star^b^										
		Hap_2	Hap_3	Hap_4	Hap_5	Hap_6	Hap_7	Hap_8	Hap_9	Hap_10	Hap_11	Hap_12
1	5-LRP-7^c^	−^c^										
2	49-**KSN**TPEILGNYL-60^d^	48–51	48–51	48–51	48–51	48–51	48–51	48–51	48–51	48–51	48–51	48–51
3	138-Y**GKRG**FITD-146^d^	139–142	139–142	139–142	139–142	139–142	139–142	139–142	139–142	139–142	139–142	139–142
4	160-VE**DNG**WGAY**NGS**WWDLSVNAYFYSV**DS**RLPETSLFYKRVP-199^d^	162–164 169–171 185–188	162–164 169–171 185–188	162–164 169–171 185–188	162–164 169–171 185–188	162–164 169–171 185–188	162–164 169–171 185–188	162–164 169–171 184–186	162–164 169–171 185–188	162–164 169–171 185–188	162–164 169–171 184–186	162–164 169–171 185–188
5	203-CLTLVDPLTGWPTDQYQNVLQCQNWQYG**PPRFTN**-236^d^	231–238	231–238	231–238	231–238	231–238	231–238	231–238	231–238	231–238	231–238	231–238

*Note*: ^a^The first and second digits are the starting position of the amino acid chain;

b 160–199 amino acid chain segments appear;

c 5–7 amino acid chains do not predict conserved B-cell epitopes in DNA Star;

d Bold letters indicate B-cell antigen epitopes conserved by amino acid chains that overlap the 2 prediction methods.

## Discussion

This study builds upon both morphological and genetic confirmation of *E. granulosus* sensu lato, focusing on the identification of B-cell antigenic epitopes in the TSP11 protein of the protoscolex of Yunnan Province and its surrounding regions. The diversity of the ND1 gene sequence presently allows the classification of *E. granulosus* into 10 genotypes (Yang *et al*., 2015) and 5 strains. Specifically, G1–G3 represent *E. granulosus* strains (*Echinococcus granulosus* sensu stricto), with G1 for the sheep strain, G2 for the Tasmanian sheep strain（this genotype is not currently recognized as valid）and G3 for the water buffalo strain (Omadang *et al*., 2024). The remaining strains, G4–G10, include the horse strain (G4 – *Echinococcus equinus*), Ortlepp's strain (G5 – *Echinococcus ortleppi*) and the Canadian strain (G6–G10) comprising the camel strain (G6), pig strain (G7), deer strain (G8), Poland strain (G9)（this genotype is not currently recognized as valid）and elk strain (G10) (Nakao *et al*., 2007;, 2013; Wassermann *et al*., 2024). The predominant genotypes observed among the *E. granulosus* samples in this study were G1 and G5, with G1 being the most prevalent. However, the differences in detection rates of G1 and G5 genotypes across various intermediate hosts such as humans, pigs, cattle and sheep did not attain statistical significance, indicating an absence of bias in host sources. However, its prevalence was slightly lower than the previously reported detection rates of 98.1 and 97.9% in Chinese populations and animals (Alvarez *et al*., 2014; Zhang *et al*., 2014). This discrepancy may be attributed to the high sensitivity of the ND1 full-gene sequence alignment utilized for genotyping in this study, resulting in 9.5% of the sample sequences being unclassified according to the published reference sequences.

The Tetraspanin (TSP) family is a crucial component of the tetraspanin-enriched membrane microdomain (TEM) superfamily, comprising 4 main subfamilies: the CD family (CD9, CD81 and CD151), the slow retinal degeneration (RDS) family (RDS-ROM), the uroplakin family (UPK1A/1B) and the CD63 family (CD63 and TSPAN31). These proteins, ranging from 200 to 350 amino acids (Hu *et al*., 2015; Xian *et al*., 2021), exhibit a structural composition that includes 2 extracellular domains known as the small extracellular loop (EC1) and large extracellular loop (EC2/LEL), an intracellular loop (transitioning from structural domain 2–3), and N- and C-terminal tails. Among the 4 highly conserved transmembrane domains (TM1–4) of TSP, the EC2/LEL structural domain, referred to as the ‘Tetraspanin Structural Web,’ serves as a binding site for numerous ligand proteins and stands out as an area of concentration for anti-TSP antibodies (Piratae *et al*., 2012; Graham *et al*., 2020; Ahmed *et al*., 2021).

TSP11 has long been recognized as a promising candidate protein for vaccine development targeting various diseases, including schistosomiasis (Tran *et al*., 2006; Cardoso *et al*., 2008; Jiang *et al*., 2010; Zhang *et al*., 2011), clonorchiasis (Kim *et al*., 2012; Chaiyadet *et al*., 2017), opisthorchiasis (Piratae *et al*., 2012; Tomii *et al*., 2019; Phumrattanaprapin *et al*., 2021), Manson's schistosomiasis (Pearson *et al*., 2012; Cheng *et al*., 2013; Curti *et al*., 2013; Jia *et al*., 2014), filariasis (Dakshinamoorthy *et al*., 2013) and pulmonary hydatid disease (Dang *et al*., 2009*a*, 2009*b*, 2012*a*, 2012*b*). Additionally, it has been explored as a potential target for detecting *Taenia solium* infection in pigs. The detection of circulating antigen TSP11 in the human body has demonstrated high sensitivity and specificity in diagnosing cysticercosis (Hancock *et al*., 2006; Moribe and Mekada, 2013). In this study, the TSP11 gene CDS length of the protoscoleces in 42 samples remained consistently at 894 bp, resulting in an amino acid chain of 298 aa. The 24 reported SNP mutations were novel, and their detection differences among strains from various hosts, including humans, pigs, cattle and sheep, were statistically insignificant. While there is a certain bias in the host source diversity of the TSP11 gene CDS sequence – with Hap_4, Hap_5 and Hap_11 sequences detected exclusively in human-derived strains, Hap_6 only in pig-derived strains, and Hap_9 and Hap_12 only in cattle-derived strains – and Hap_7, Hap_8 and Hap_10 exclusively present in sheep-derived strains, these haplotypes are largely situated at the evolutionary distant end. Hap_2 and Hap_3, considered earlier ancestors, are detectable in parasitic strains from all 4 intermediate hosts (Fig. 3), suggesting the rationale for selecting sequences from Hap_2 and Hap_3 for the detection of antigens in the 4 intermediate hosts.

A comprehensive evaluation of B-cell antigenic epitopes for the 42 amino acid chains, considering hydrophilicity (Ponomarenko *et al*., 2011), accessibility (Allcorn and Martin, 2002), flexibility (Li *et al*., 2016) and antigenicity (Resende *et al*., 2012), reveals 6 peptide regions, namely 49-KSN-51, 139-GKRG-142, 162-DNG-164, 169-NGS-171, 185-DS-186 and 231-PPRFTN-236, distributed across strains from different species and haplotypes. These 6 peptide chains exhibit robust conservation. Among the 24 SNPs, only the c.492 C > T synonymous mutation appears in the second amino acid (G164G) codon within the 162-DNG-171 peptide chain. However, among these 6 B-cell antigenic epitopes, based on the findings of Wang *et al*. (Wang *et al*., 2020) and Xian Jinwen (Xian *et al*., 2021) regarding the strong antigenicity and immunogenicity of the 227-WQYGPPRFTNGAHN-240 peptide chain and its extracellular loop (LEL) region, and considering the principle that B-cell antigenic epitopes are preferably composed of 5–15 amino acid residues (Gong, 2016), this study supports the utilization of the 231-PPRFTN-236 peptide chain as an immunodiagnostic tool for developing a broadly effective immune diagnosis for *E. granulosus* protoscoleces infection in different hosts.

This study marks a significant milestone as it successfully obtained the gene sequencing sequences of the 4 exons of the TSP11 gene from within the epidemic region of Yunnan Province, *E. granulosus* sensu lato. This achievement contributes substantially to the augmentation of shared data within GenBank. Furthermore, the study delves into the mutation sites and haplotypes of the TSP11 gene, offering valuable insights into the potential applications of the tetraspanin family in addressing *E. granulosus*. Nevertheless, certain limitations exist. The lack of specific township localization in the sample sources restricts the ability to make precise comparisons of the prevalence in different regions. Additionally, due to spatial constraints, this paper does not extend to the validation of the diagnostic antigen's efficacy selected from the TSP11 gene. Moving forward, the research group plans to undertake pertinent studies to evaluate and substantiate the diagnostic antigen's effectiveness for *E. granulosus*.

## Conclusion

This study sheds light on prevalent intermediate hosts in the endemic regions of Yunnan Province, primarily infected with G1 and G5 genotypes of *E. granulosus* protoscoleces. The report introduces 24 novel nucleotide peptide sites and uncovers 11 haplotypes for the TSP11 transmembrane protein in Yunnan, all representing mutated forms. Proposing 6 potential B-cell antigenic epitopes in TSP11, the study maintains consistency among different intermediate hosts and haplotypes, laying the groundwork for considering TSP11 protein as a candidate antigen for diagnosing different species of *E. granulosus* protoscolecess, offering theoretical support for the diagnosis of *E. granulosus*.

## Supporting information

Xu et al. supplementary material 1 Xu et al. supplementary material

Xu et al. supplementary material 2 Xu et al. supplementary material

## Data Availability

Availability of Data: The materials and data related to this study are currently unavailable.

## Supplementary material

The supplementary material for this article can be found at https://doi.org/10.1017/S0031182024000726.
