# Supplementary material for: Molecular characterization and B-cell epitope analysis of the TSP11 gene in Echinococcus infection strains from Yunnan Province
Source: Parasitology. 2024 Nov 12;151(10):1108–17. doi: 10.1017/S0031182024000726 (PMC11894004; doi:10.1017/S0031182024000726)
Supplement: Xu et al. supplementary material 1 — Xu et al. supplementary material [file S0031182024000726sup001.docx]

**Supplementary Material 1**

| **Information table of Echinococcus granulosus (diseased organ specimen )** | | | | | | | | |
| --- | --- | --- | --- | --- | --- | --- | --- | --- |
| Order | geographic coordinates | sampling sites | | sampling date | host | lesion location | The results of pathogenic detection of the lesion | Experiment number |
|  |  | State and city | County (district, city) |  |  |  |  |  |
| 1 | E99.28,N27.18 | Diqing Tibetan Autonomous Prefecture | Weixi Lisu Autonomous County | 2016 | pig | liver | Double-walled structure, inside can be seen powdery inner capsule, the cyst fluid is bright and slightly turbidous, can be seen procephala | 2016-1 |
| 2 | E99.71,N27.82 | Diqing Tibetan Autonomous Prefecture | Shangri-La City | 2017 | cow | liver | Double-walled structure, the inner capsule of powdery skin can be seen, and the liquid of the capsule is clear and slightly turbid | 1 |
| 3 | E99.71,N27.82 | Diqing Tibetan Autonomous Prefecture | Shangri-La City | 2017 | cow | liver | Double-walled structure with a powdery inner capsule containing jelly-like contents | 2 |
| 4 | E99.71,N27.82 | Diqing Tibetan Autonomous Prefecture | Shangri-La City | 2017 | cow | liver | Double-walled structure, inside can be seen powder-like capsule, capsule filled with calcifications | 6 |
| 5 | E99.71,N27.82 | Diqing Tibetan Autonomous Prefecture | Shangri-La City | 2017 | cow | liver | Double-walled, powdery inner capsule with turbidous fluid and protocephalic segment | 13 |
| 6 | E99.71,N27.82 | Diqing Tibetan Autonomous Prefecture | Shangri-La City | 2017 | cow | lung | Double-walled, powdery inner capsule with turbidous fluid and protocephalic segment | 15 |
| 7 | E99.71,N27.82 | Diqing Tibetan Autonomous Prefecture | Shangri-La City | 2017 | cow | lung | Double-walled, powdery inner capsule with turbidous fluid and protocephalic segment | 31 |
| 8 | E99.71,N27.82 | Diqing Tibetan Autonomous Prefecture | Shangri-La City | 2017 | cow | liver | Double-walled structure, inside can be seen powder-like capsule, capsule filled with calcifications | 39 |
| 9 | E98.86,N25.84 | Nujiang of the Lisu Autonomous Prefecture | Lushui City | 2017 | pig | liver | Double-walled structure, inside can be seen powdery capsule, capsule fluid clear and bright | 21 |
| 10 | E98.86,N25.84 | Nujiang of the Lisu Autonomous Prefecture | Lushui City | 2017 | pig | liver | Double-walled structure, inside can be seen powdery capsule, capsule fluid clear and bright | 25 |
| 11 | E99.28,N27.18 | Diqing Tibetan Autonomous Prefecture | Shangri-La City | 2017 | pig | liver | Double-walled structure with powdery capsule | 33 |
| 12 | E99.28,N27.18 | Diqing Tibetan Autonomous Prefecture | Weixi Lisu Autonomous County | 2018 | cow | liver | Double-walled structure, inside can be seen powdery capsule, capsule fluid clear and bright | 11 |
| 13 | E99.71,N27.82 | Diqing Tibetan Autonomous Prefecture | Shangri-La City | 2018 | cow | liver | Double-walled structure, inside can be seen powder-like capsule, capsule filled with calcifications | 17 |
| 14 | E99.71,N27.82 | Diqing Tibetan Autonomous Prefecture | Shangri-La City | 2018 | cow | lung | Double-walled, powdery inner capsule with turbidous fluid and protocephalic segment | 41 |
| 15 | E99.28,N27.18 | Diqing Tibetan Autonomous Prefecture | Weixi Lisu Autonomous County | 2018 | sheep | liver | Double-walled structure, inside can be seen powdery capsule, capsule fluid clear and bright | 9 |
| 16 | E103.89,N27.75 | Zhaotong City | Daguan county | 2018 | pig | liver | Double-walled structure, the inner capsule of powdery skin can be seen, and the liquid of the capsule is clear and slightly turbid | 23 |
| 17 | E103.89,N27.75 | Zhaotong City | Daguan county | 2018 | pig | liver | Double-walled structure, the inner capsule of powdery skin can be seen, and the liquid of the capsule is clear and slightly turbid | 26 |
| 18 | E103.89,N27.75 | Zhaotong City | Daguan county | 2018 | pig | liver | Double-walled structure, the inner capsule of powdery skin can be seen, and the liquid of the capsule is clear and slightly turbid | 27 |
| 19 | E103.89,N27.75 | Zhaotong City | Daguan county | 2018 | pig | liver | Double-walled structure, the inner capsule of powdery skin can be seen, and the liquid of the capsule is clear and slightly turbid | 29 |
| 20 | E99.95,N26.11 | Dali Bai Autonomous prefecture | Eryuan county | 2018 | pig | liver | Double-walled structure, inside can be seen powdery capsule, capsule fluid clear and bright | 34 |
| 21 | E99.95,N26.11 | Dali Bai Autonomous prefecture | Eryuan county | 2018 | pig | liver | Double-walled structure, inside can be seen powdery capsule, capsule fluid clear and bright | 36 |
| 22 | E99.95,N26.11 | Dali Bai Autonomous prefecture | Eryuan county | 2018 | pig | liver | Double-walled structure, inside can be seen powdery capsule, capsule fluid clear and bright | 37 |
| 23 | E103.89,N27.75 | Zhaotong City | Daguan county | 2018 | pig | liver | Double-walled structure, the inner capsule of powdery skin can be seen, and the liquid of the capsule is clear and slightly turbid | 49 |
| 24 | E99.71,N27.82 | Diqing Tibetan Autonomous Prefecture | Shangri-La City | 2019 | cow | lung | Double-walled structure with powdery capsule | 2020-3 |
| 25 | E102.37,N23.36 | Honghe Autonomous Prefecture | Honghe County | 2019 | sheep | liver | Double-walled structure with powdery capsule | 2020-2 |
| 26 | E98.86,N25.84 | Nujiang of the Lisu Autonomous Prefecture | Lushui City | 2019 | pig | liver | Double-walled structure with powdery capsule | 2020-4 |
| 27 | E99.71,N27.82 | Diqing Tibetan Autonomous Prefecture | Shangri-La City | 2020 | cow | liver,lung | Double-walled, powdery inner capsule with turbidous fluid and protocephalic segment | 2-1 |
| 28 | E99.71,N27.82 | Diqing Tibetan Autonomous Prefecture | Shangri-La City | 2020 | cow | lung | Double-walled structure with powdery capsule | 2-3 |
| 29 | E99.71,N27.82 | Diqing Tibetan Autonomous Prefecture | Shangri-La City | 2020 | cow | liver | Double-walled structure with powdery capsule | 2-4 |
| 30 | E99.91,N26.54 | Dali Bai Autonomous prefecture | Jianchuan county | 2017 | person | liver | Double-walled structure with powdery capsule | H1 |
| 31 | E99.91,N26.54 | Dali Bai Autonomous prefecture | Jianchuan county | 2018 | person | liver | Double-walled structure with powdery capsule | H2 |
| 32 | E100.24,N26.82 | Lijiang City | Yulong county | 2016 | person | liver | Double-walled structure with powdery capsule | H3 |
| 33 | E100.24,N26.82 | Lijiang City | Yulong county | 2018 | person | liver | Double-walled structure with powdery capsule | H4 |
| 34 | E99.28,N27.18 | Diqing Tibetan Autonomous Prefecture | Weixi Lisu Autonomous County | 2019 | person | liver | Double-walled structure with powdery capsule | H5 |
| 35 | E101.96,N30.05 | sichuan province | garze zang natingality prefecture | 2016 | person | liver | Double-walled structure with powdery capsule | H6 |
| 36 | E101.96,N30.05 | sichuan province | garze zang natingality prefecture | 2016 | person | liver | Double-walled structure with powdery capsule | H7 |
| 37 | E101.96,N30.05 | sichuan province | garze zang natingality prefecture | 2016 | person | liver | Double-walled structure with powdery capsule | H8 |
| 38 | E101.96,N30.05 | sichuan province | garze zang natingality prefecture | 2016 | person | liver | Double-walled structure with powdery capsule | H9 |
| 39 | E99.91,N26.54 | Dali Bai Autonomous prefecture | Jianchuan county | 2015 | person | liver | Double-walled structure with powdery capsule | H10 |
| 40 | E99.91,N26.54 | Dali Bai Autonomous prefecture | Jianchuan county | 2018 | person | liver | Double-walled structure with powdery capsule | 38 |
| 41 | E100.24,N26.82 | Lijiang City | Yulong county | 2018 | person | liver | Double-walled structure with powdery capsule | 28 |
| 42 | E99.28,N27.18 | Diqing Tibetan Autonomous Prefecture | Weixi Lisu Autonomous County | 2019 | person | liver | Double-walled structure with powdery capsule | 12 |
