# Supplementary material for: Molecular characterization and B-cell epitope analysis of the TSP11 gene in Echinococcus infection strains from Yunnan Province
Source: Parasitology. 2024 Nov 12;151(10):1108–17. doi: 10.1017/S0031182024000726 (PMC11894004; doi:10.1017/S0031182024000726)
Supplement: Xu et al. supplementary material 2 — Xu et al. supplementary material [file S0031182024000726sup002.docx]

**Supplementary Material 2**

**B-cell epitope prediction of TSP11 amino acid chain in Echinococcus granulosus infected with different intermediate hosts**

1. **IEDB online forecast chart.**

**
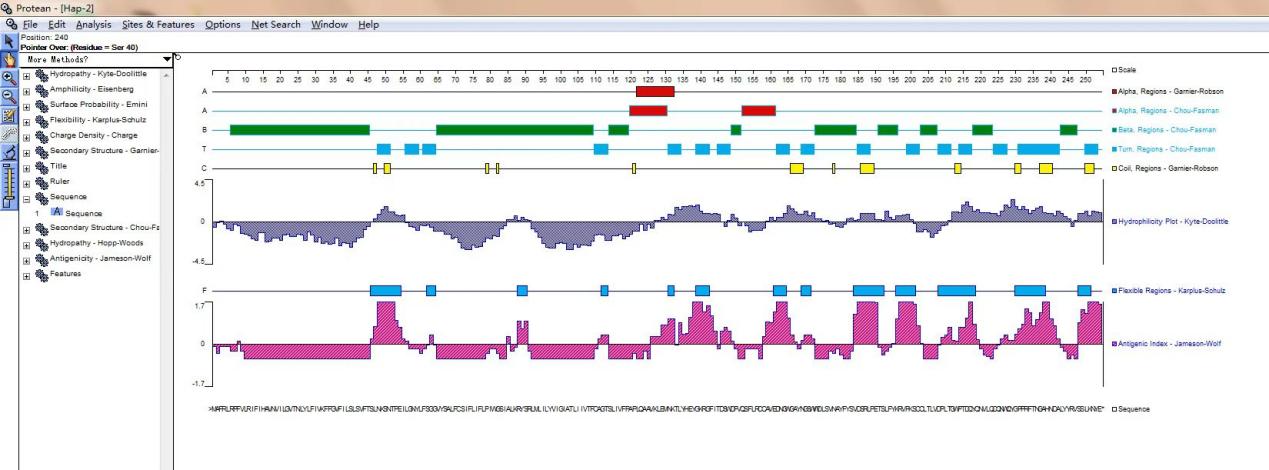

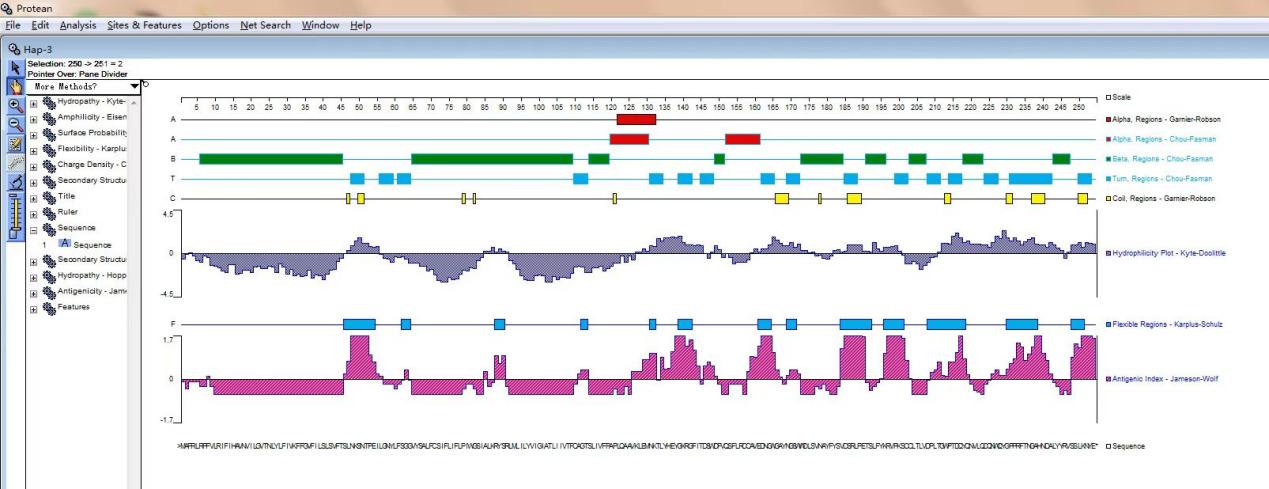

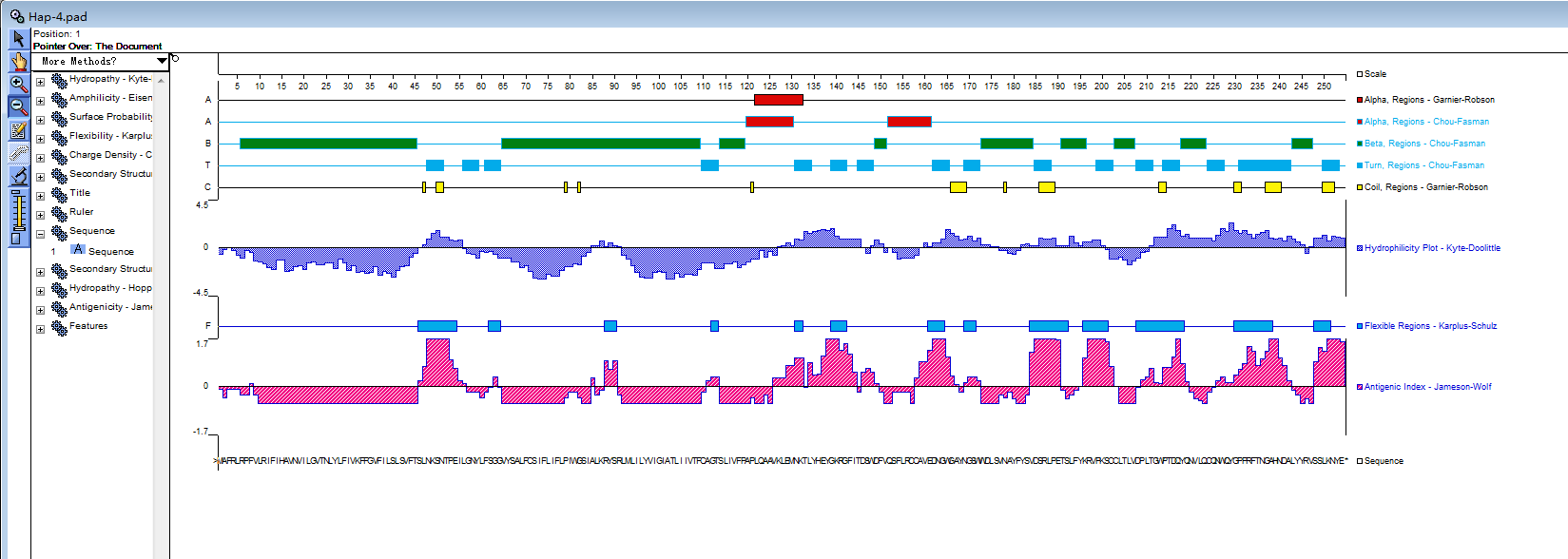

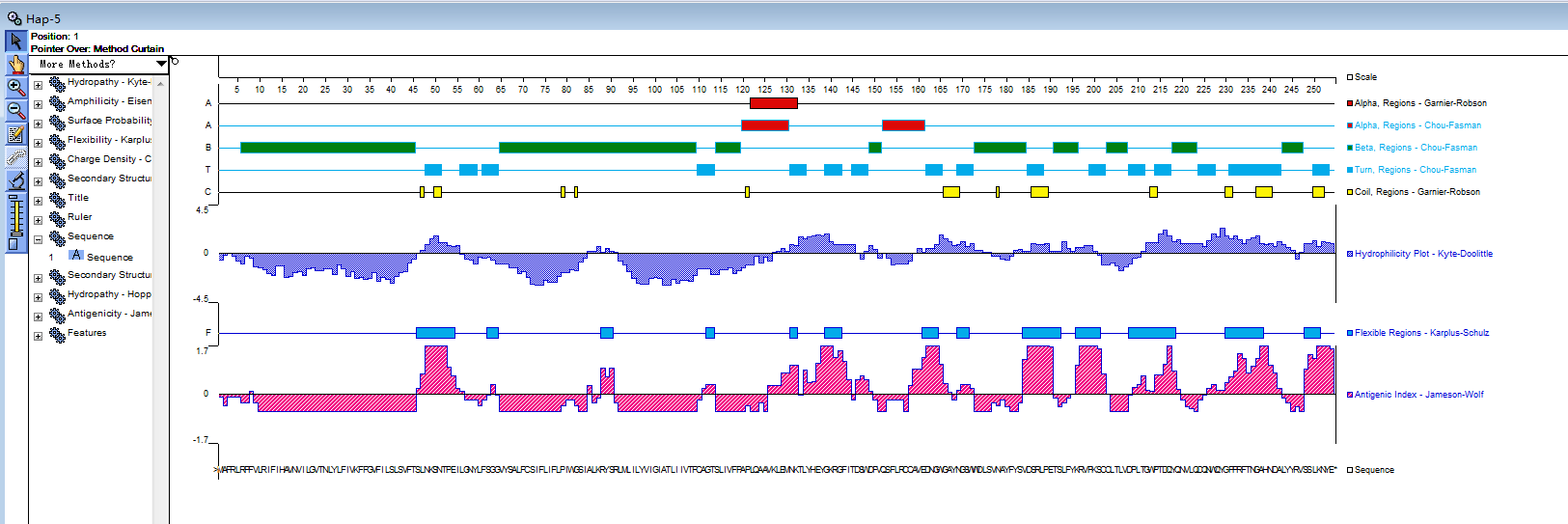

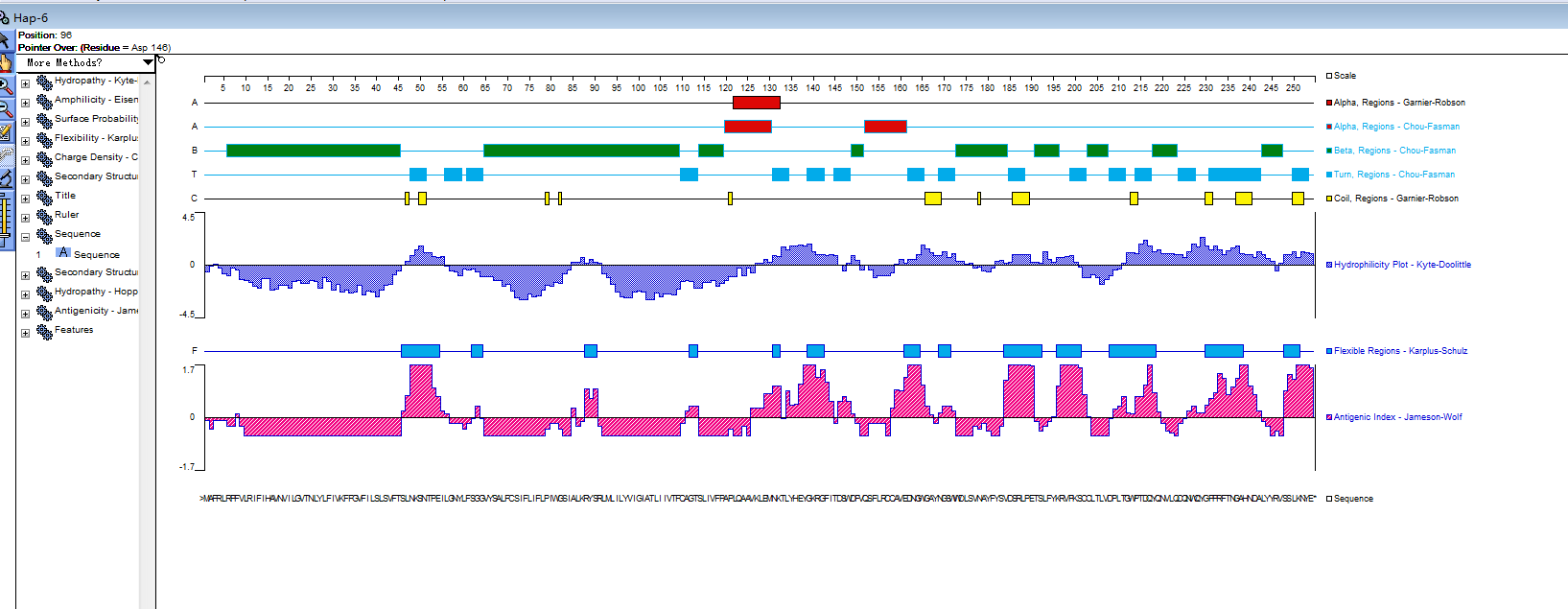

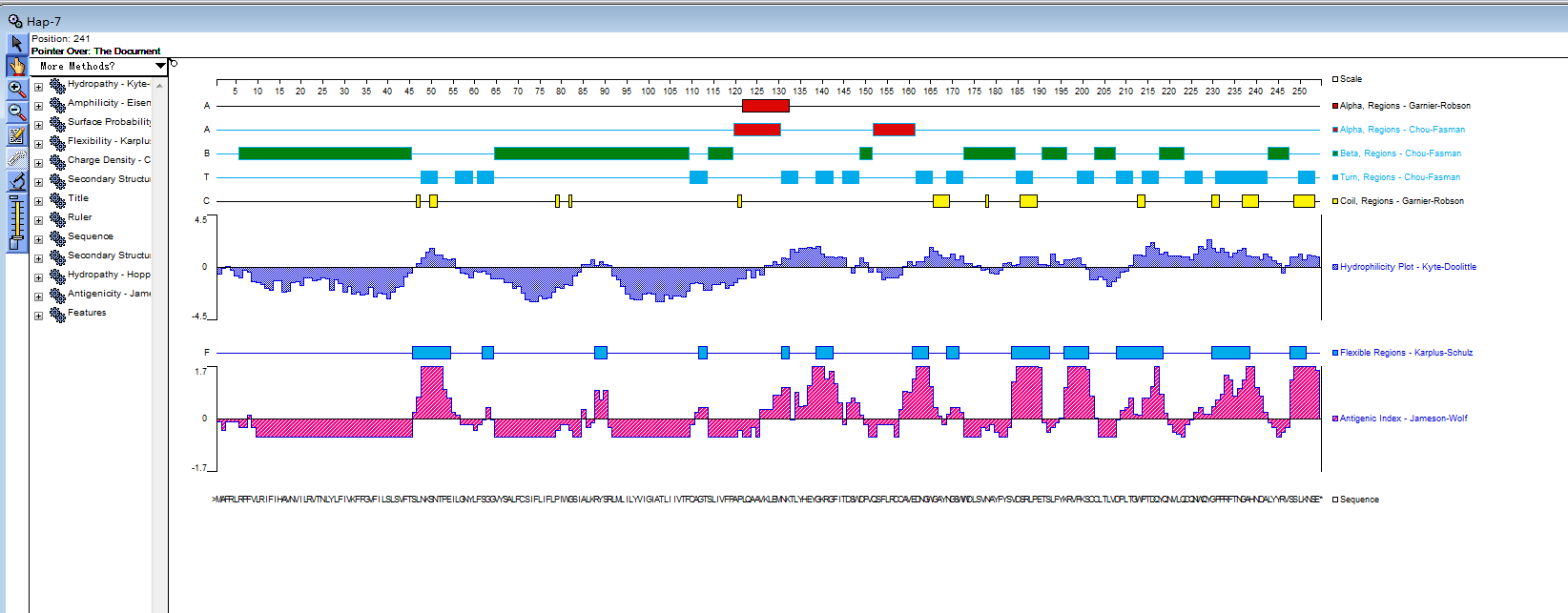

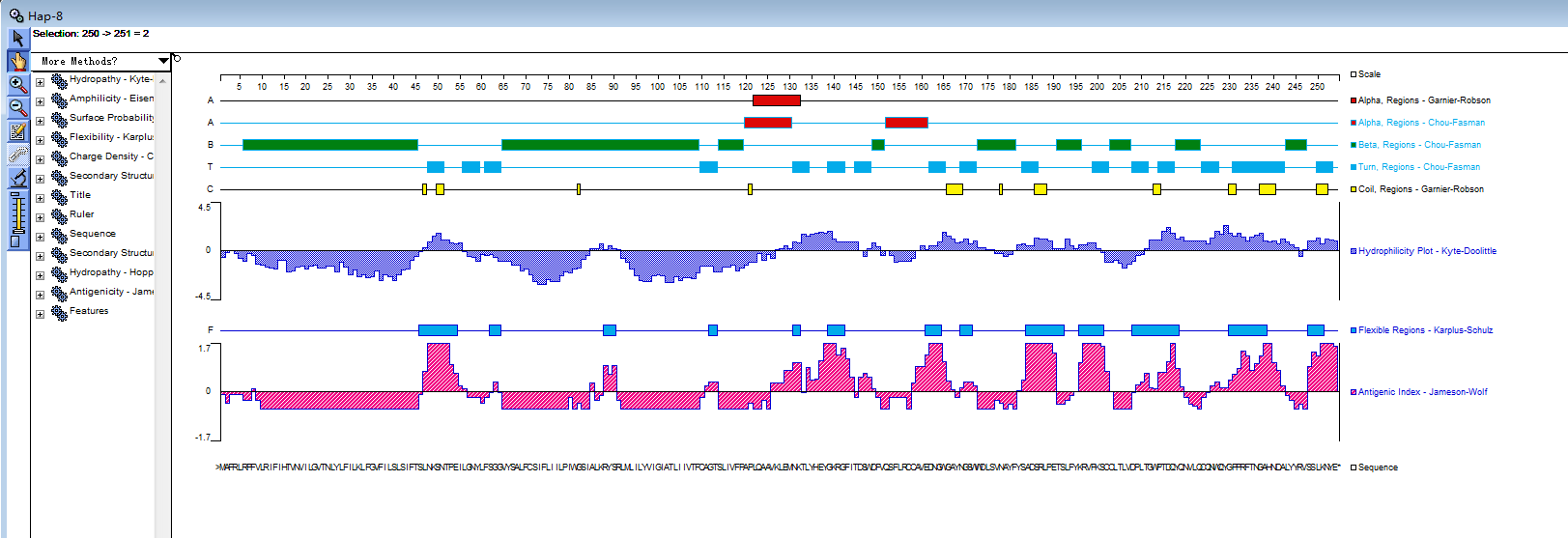

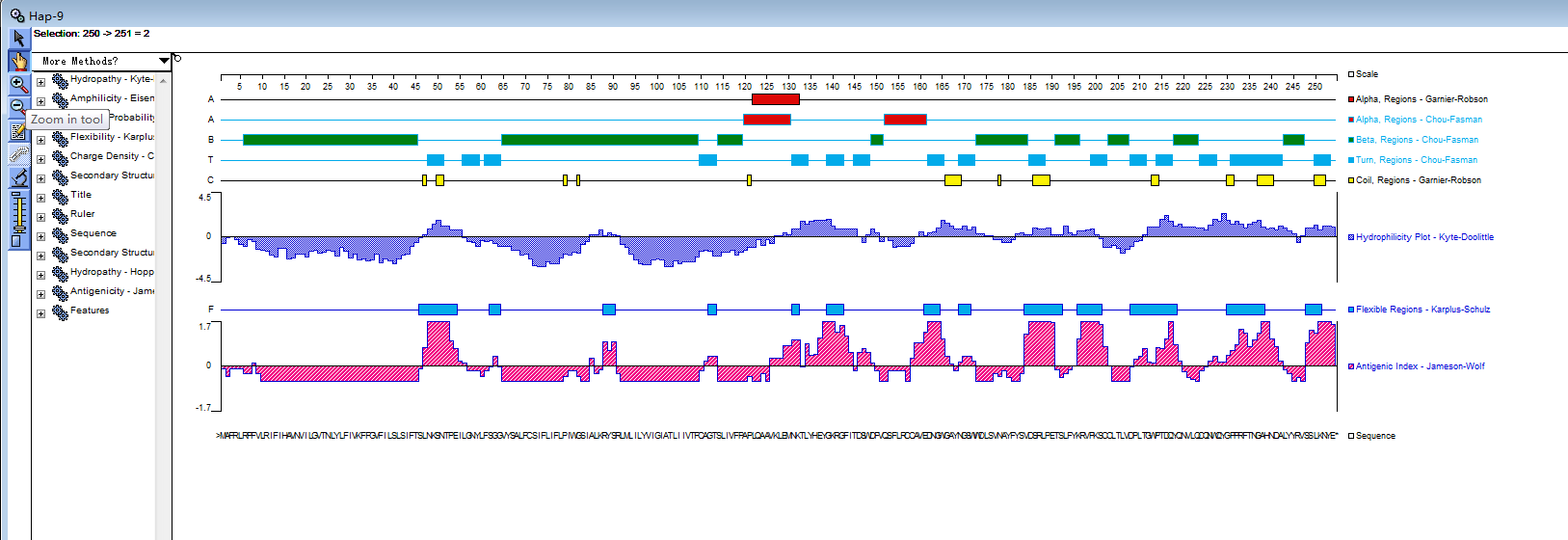

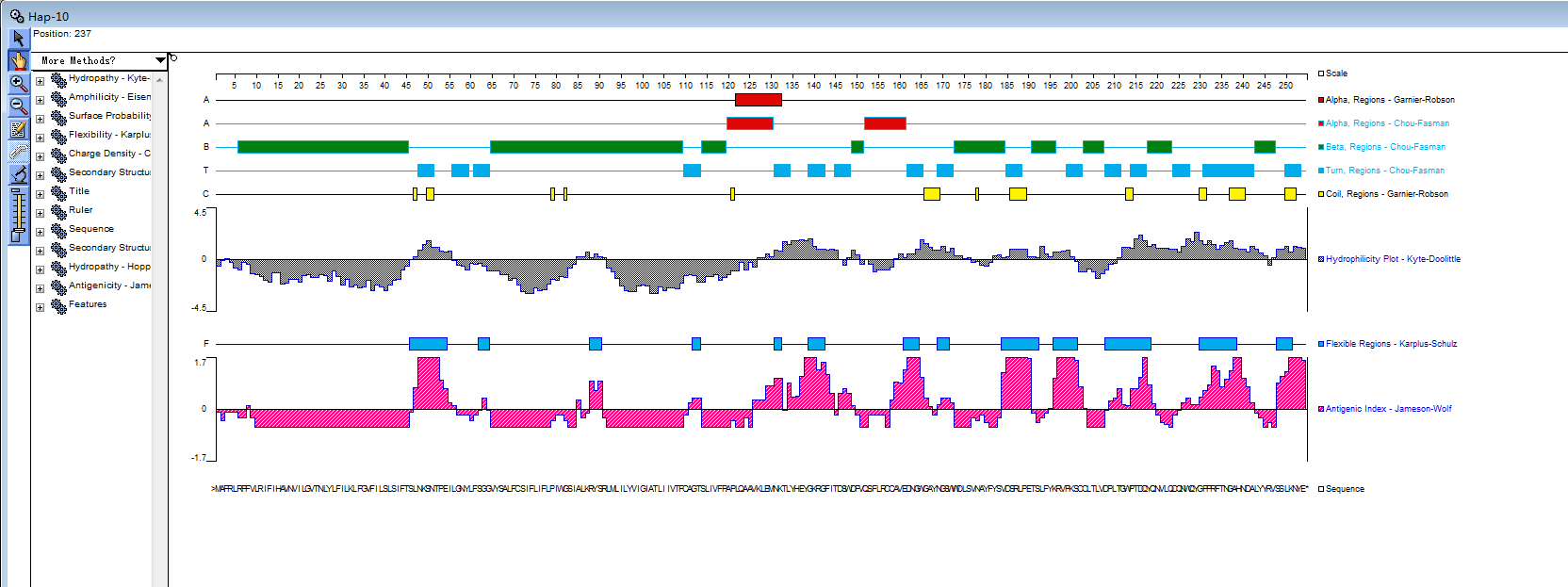

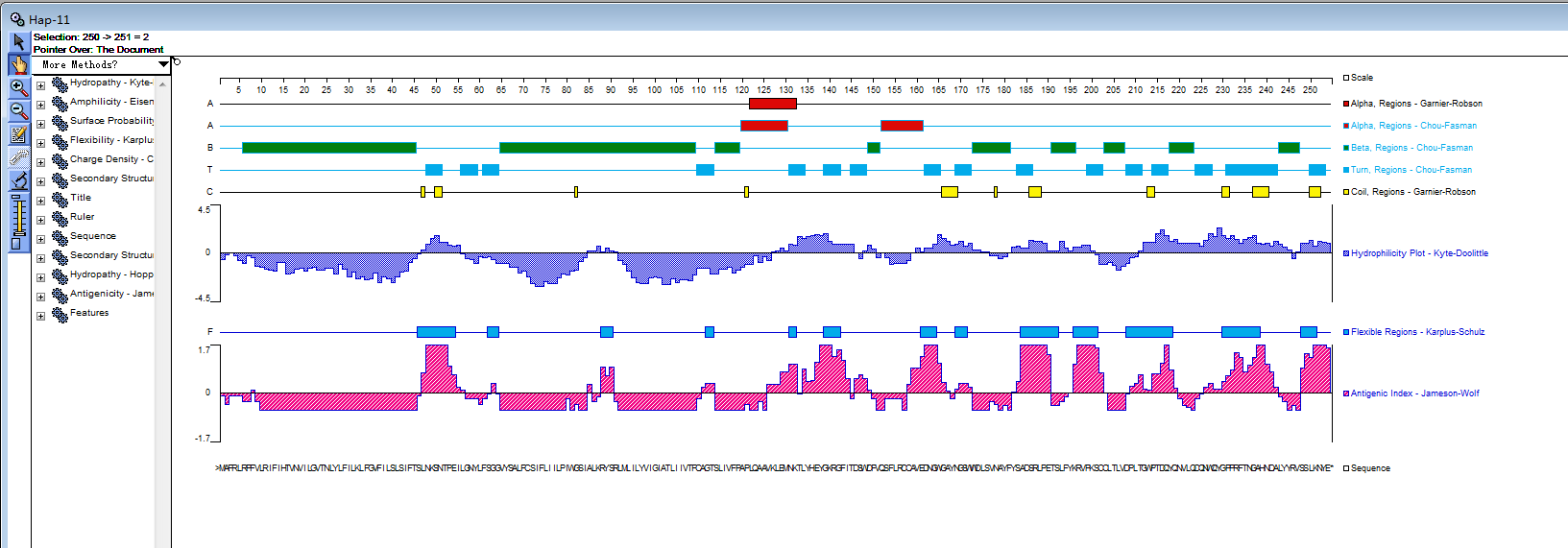

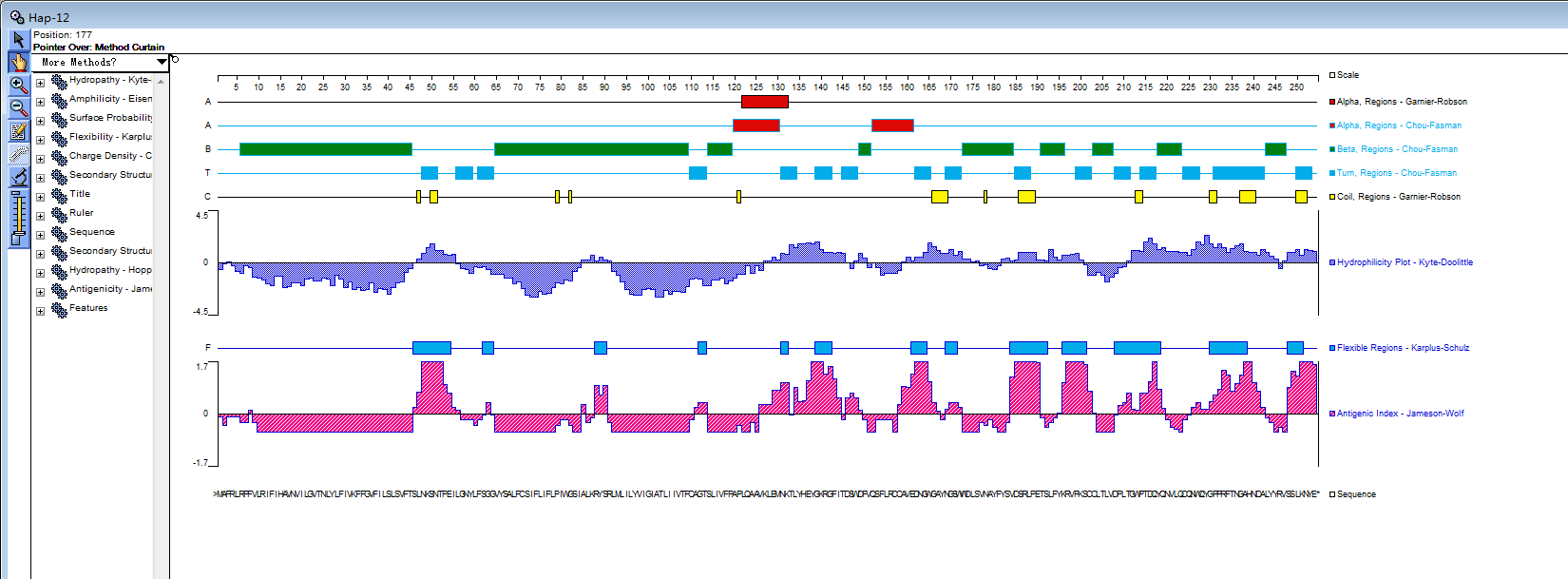
**

**(2) Prediction diagram of "Protean" module of DNA Star software.**

**
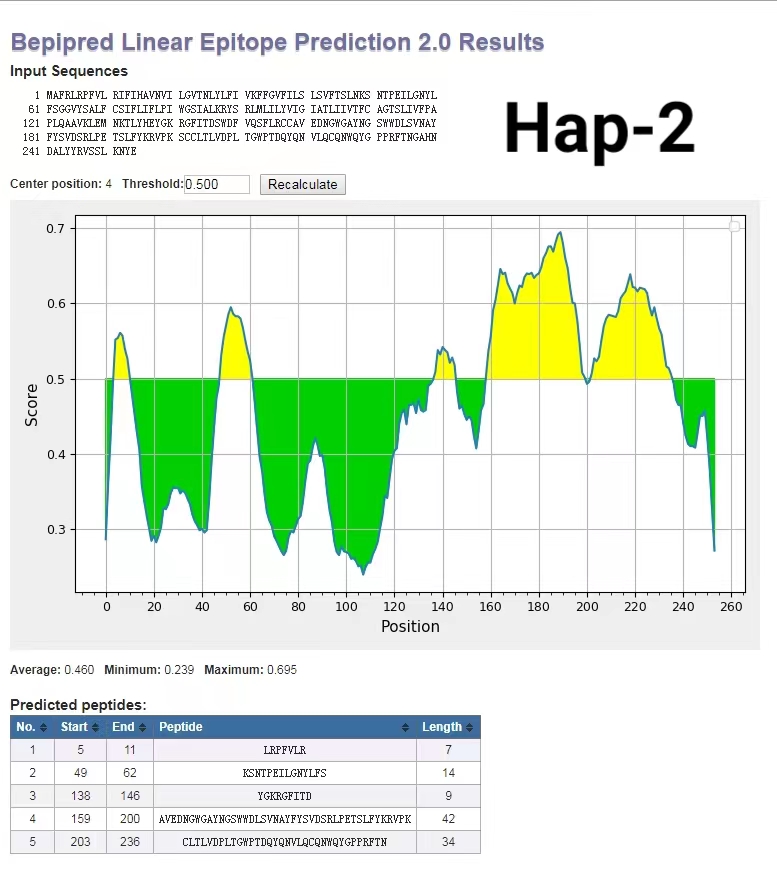

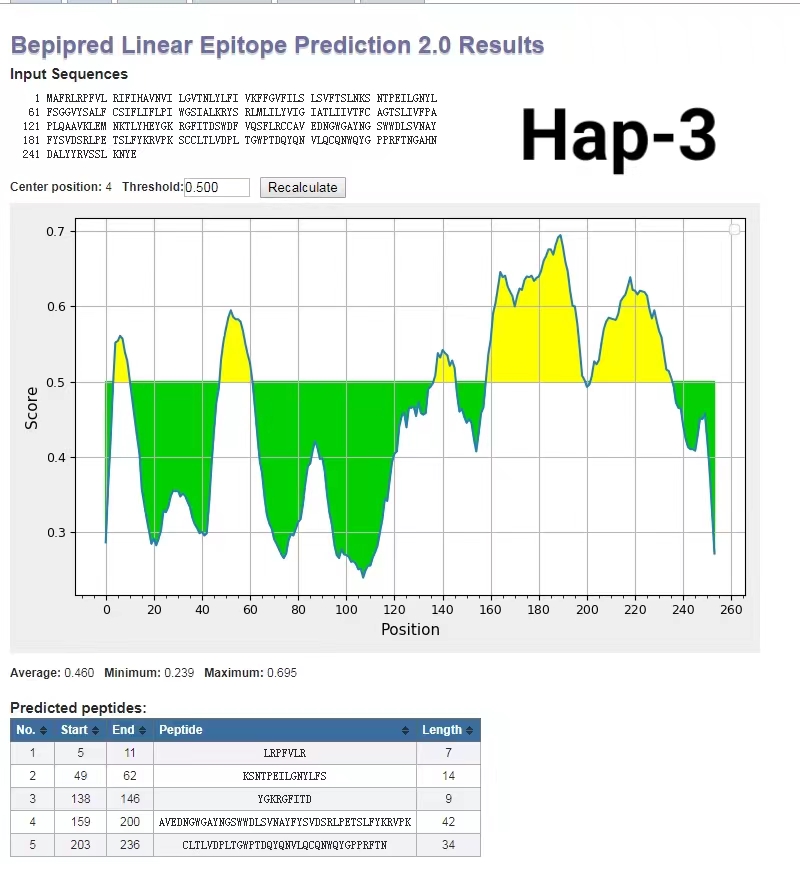

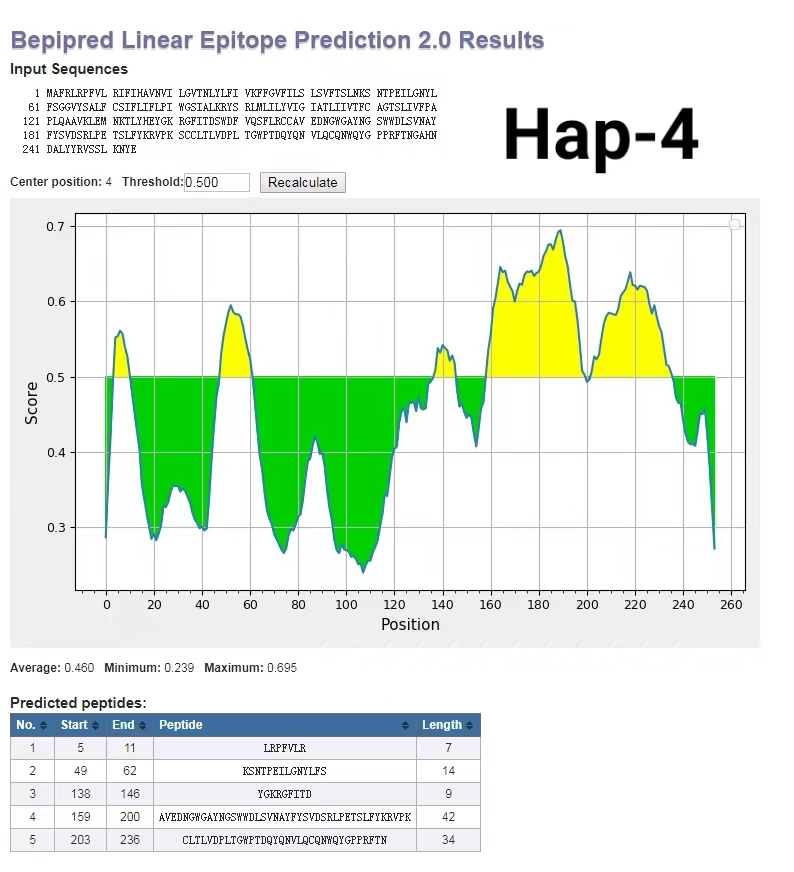

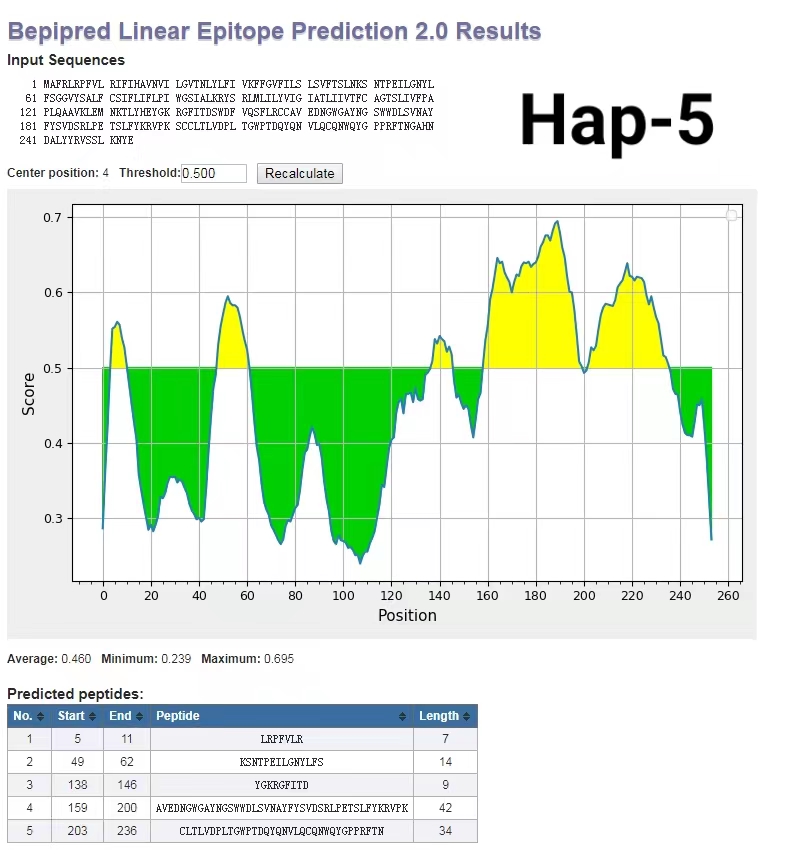

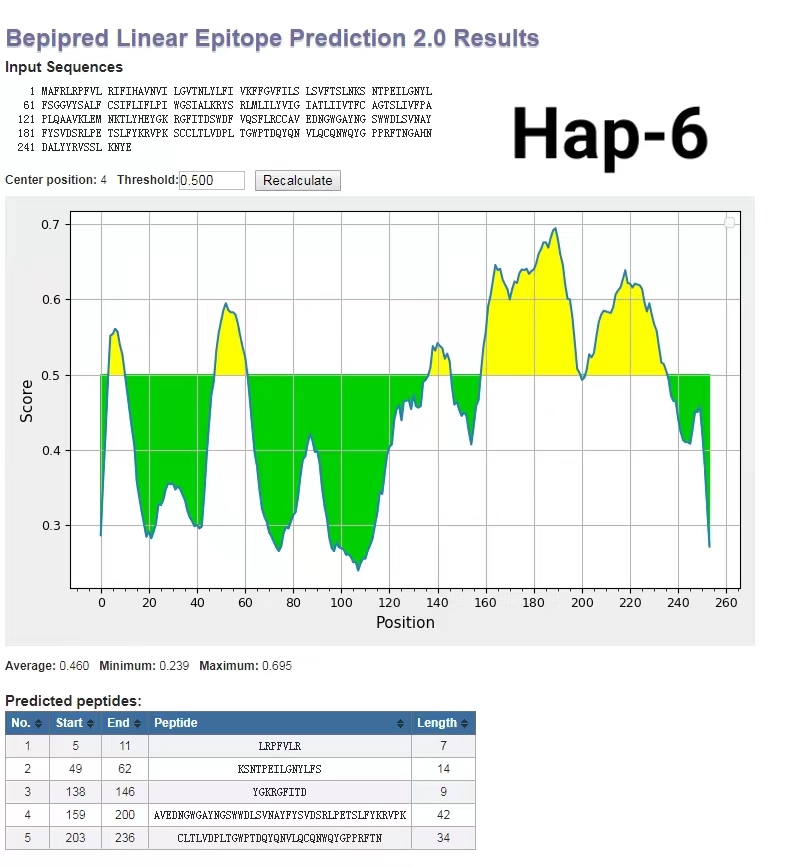

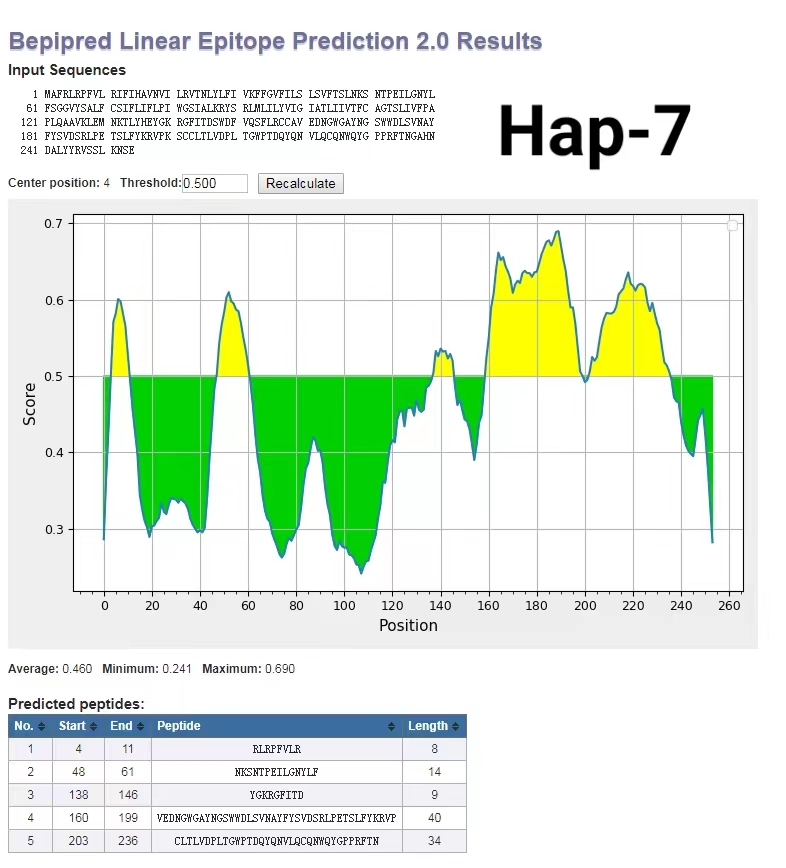

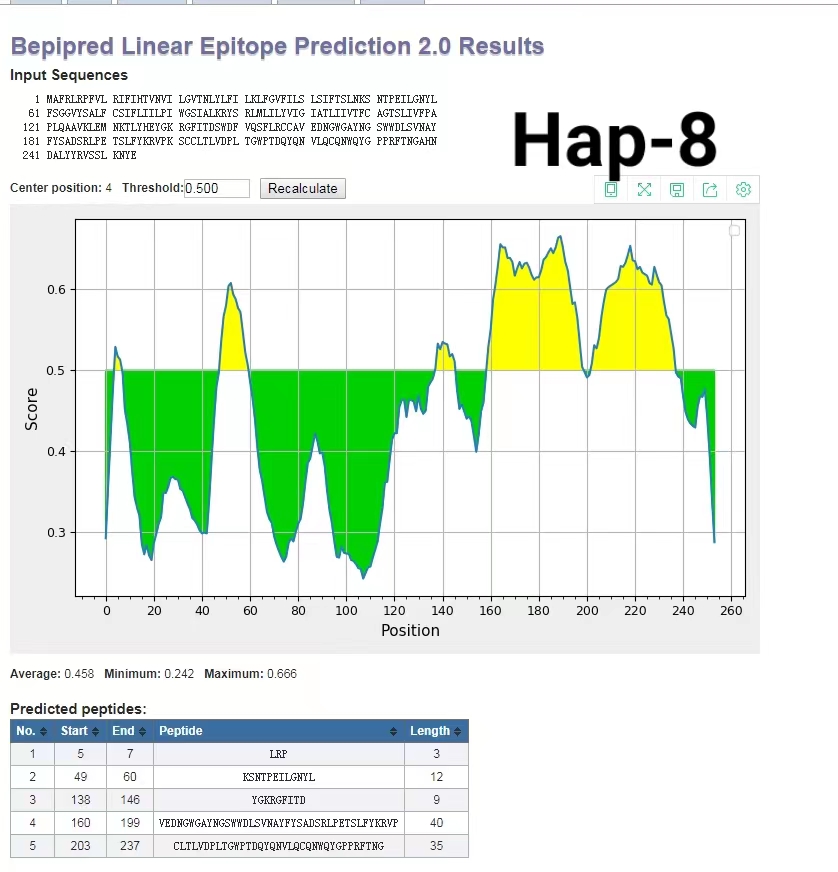

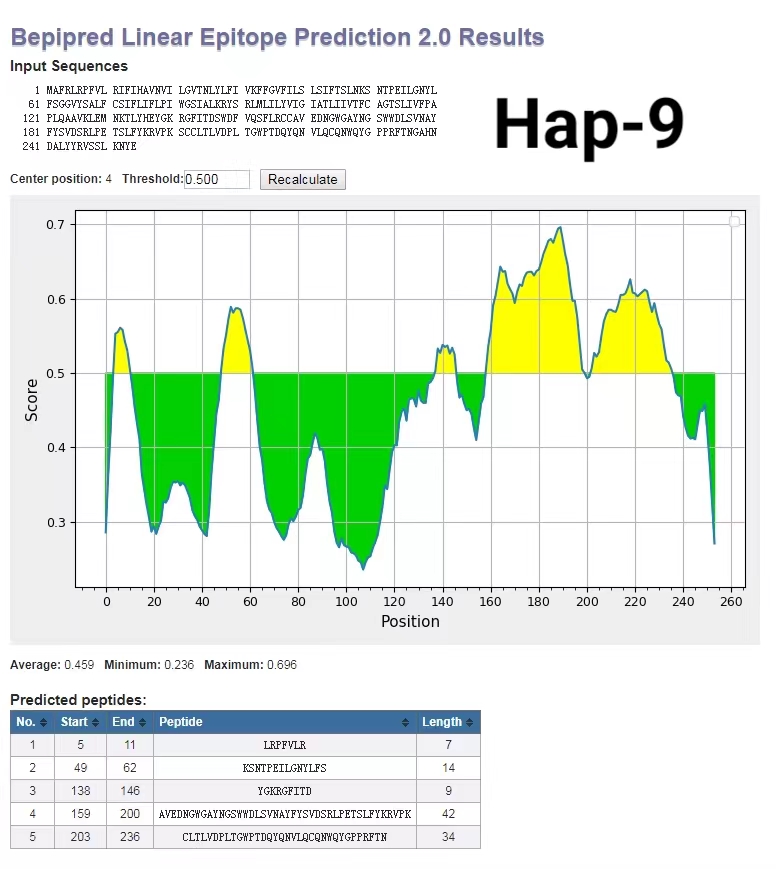
**

**
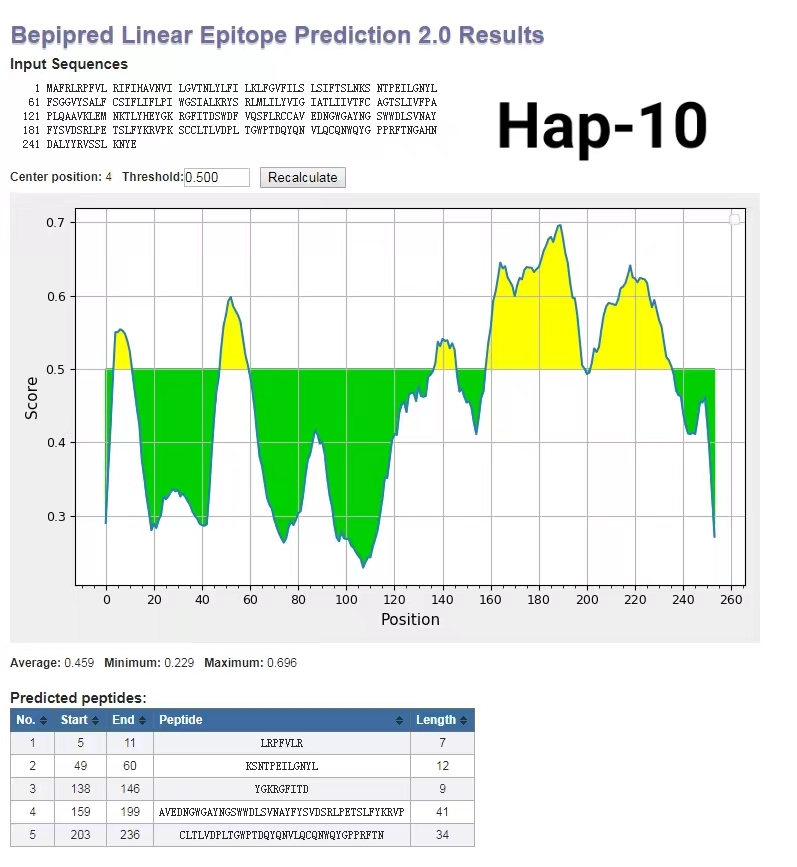

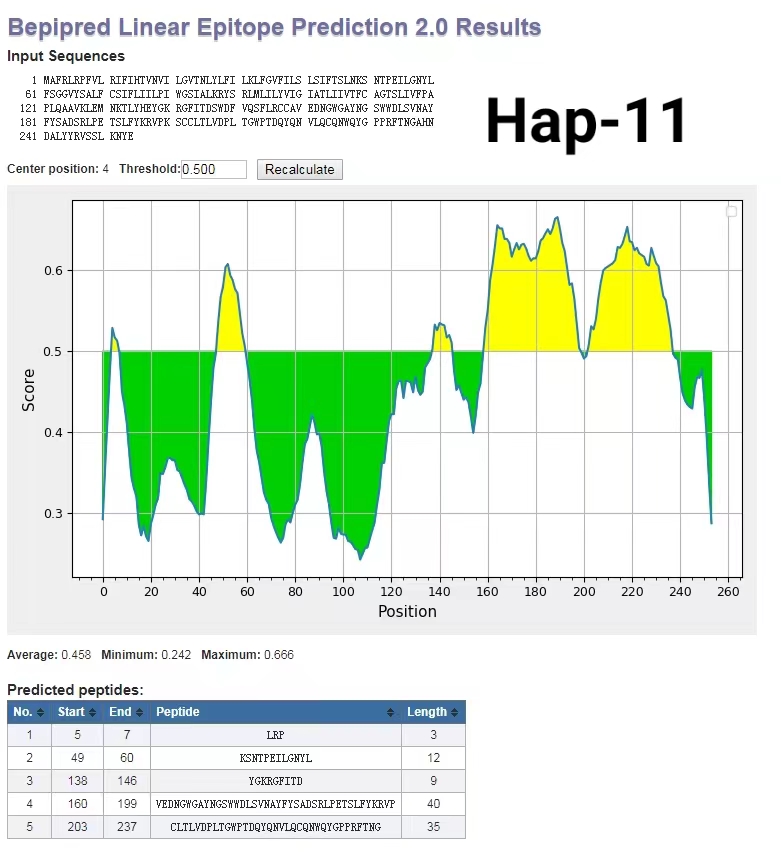
**

**
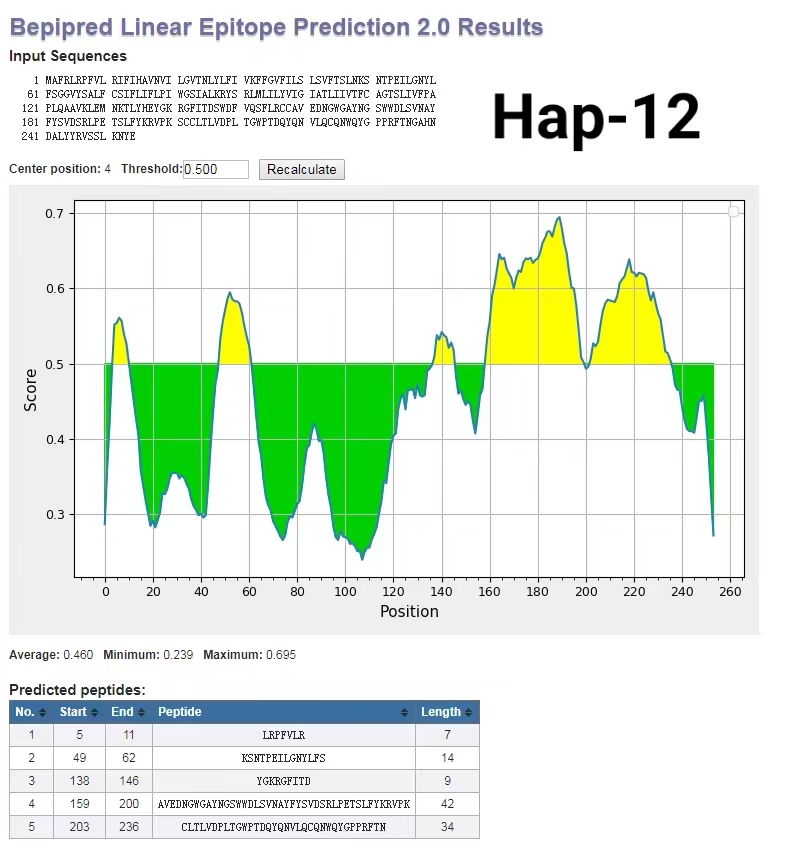
**
